# Artificial Intelligence in Pancreatobiliary Endoscopy: Current Advances, Opportunities, and Challenges

**DOI:** 10.3390/jcm14217519

**Published:** 2025-10-23

**Authors:** Aastha V. Bharwad, Rohan Ahuja, Pragya Jain, Vaibhav Wadhwa

**Affiliations:** 1 Department of Gastroenterology, University of Texas Health Science Center, Houston, TX 77030, USA; aastha.v.bharwad@uth.tmc.edu (A.V.B.); rohan.ahuja@uth.tmc.edu (R.A.); 2 Department of Internal Medicine, Baptist Hospital of Southeast Texas, Beaumont, TX 77701, USA; pragyajainmd@gmail.com

**Keywords:** artificial intelligence, pancreatobiliary endoscopy, endoscopic ultrasound, ERCP, cholangioscopy, deep learning, machine learning, computer-assisted diagnosis

## Abstract

Pancreaticobiliary endoscopy, encompassing endoscopic ultrasound (EUS), endoscopic retrograde cholangiopancreatography (ERCP), and digital single-operator cholangioscopy (DSOC), is essential for diagnosing and managing pancreatic and biliary diseases. However, these procedures are limited by operator dependency, variable diagnostic accuracy, and technical complexity. Artificial intelligence (AI), particularly through machine learning (ML) and deep learning (DL), has emerged as a promising tool to address these challenges. Early studies show that AI can enhance lesion detection, improve differentiation of pancreatic masses, classify cystic lesions, and aid in diagnosing malignant biliary strictures. AI has also been used to predict post-ERCP pancreatitis risk and reduce radiation exposure during ERCP. Despite this promise, current AI models are largely experimental—limited by small, single-center datasets, lack of external validation, and no FDA-approved systems for these indications. Major barriers include inconsistent data acquisition, limited interoperability across hardware platforms, and integration into real-time workflows. Future progress depends on multicenter data sharing, standardized imaging protocols, interpretable AI design, and regulatory pathways for model deployment and updates. AI can be developed as a valuable partner to endoscopists, enhancing diagnostic accuracy, reducing complications, and supporting more efficient, personalized care in pancreaticobiliary endoscopy.

## 1. Introduction

Pancreaticobiliary endoscopy plays a pivotal role in the diagnosis and management of a wide spectrum of pancreatic and biliary diseases through advanced diagnostic modalities such as endoscopic ultrasound (EUS), endoscopic retrograde cholangiopancreatography (ERCP), and cholangioscopy. Though these techniques have significantly advanced clinical capabilities, they remain constrained by several limitations including a steep learning curve, high dependence on operator expertise, and inherent variability in diagnostic accuracy [1,2]. Such challenges increase the risk of misdiagnosis or missed pathology, thereby impacting clinical outcomes. For example, EUS requires substantial training and is susceptible to inter-operator variability, often resulting in the under-detection of subtle lesions [3]. ERCP is technically demanding, particularly due to difficulties in bile duct cannulation [4], while cholangioscopy faces notable challenges in visualizing extrinsic strictures and distal biliary lesions [5]. Moreover, the diagnostic performance of digital single-operator cholangioscopy (DSOC) remains limited, owing to low sampling yields and the absence of standardized classification systems for distinguishing benign from malignant strictures [6].

Artificial intelligence (AI), initially developed as a branch of computer science, aims to create systems capable of tasks requiring human intelligence. A key area within AI is machine learning (ML), where algorithms learn from training data to carry out specific functions such as pattern recognition. These ML models are trained on large datasets and then validated using separate datasets to evaluate their predictive performance [1,7]. An advanced subset of ML is deep learning (DL), which uses artificial neural networks (ANNs) with multiple layers to automatically extract complex features from raw data. This layered approach allows DL models to recognize intricate patterns and make highly accurate predictions, often with minimal human intervention [1,3]. Many DL algorithms like convolutional neural networks (CNNs), recurrent neural networks (RNNs), multilayer perceptron (MLP), generative adversarial networks (GANs), and deep belief networks (DBNs) have shown promise in oncology by enabling accurate image recognition, segmentation, and predictive modeling [8].

In the field of medicine, AI is generally divided into two main branches: virtual and physical. The virtual branch encompasses ML and DL applications, while the physical branch includes medical robotics and smart devices [7]. Together, these AI technologies are transforming healthcare by enabling the analysis of vast and complex datasets with increasing precision and reduced human oversight.

While AI has seen rapid adoption in luminal gastrointestinal (GI) endoscopy, its application in pancreaticobiliary endoscopy is still in the early stages of development. This gap may be attributed to integration of multiple complex imaging modalities—such as ultrasound, fluoroscopy, and endoscopy—which increases the difficulty of developing effective AI models, and the relatively low volume of pancreaticobiliary procedures compared to general endoscopy, which limits the availability of training data [3]. Preliminary studies have explored AI applications across ERCP, EUS, and DSOC, focusing on both anatomical landmark recognition and pathology classification. For example, machine learning (ML) algorithms have been employed to identify key structures such as the ampulla during ERCP, as well as the bile duct, pancreas, and portal vein confluence during EUS [9]. Furthermore, AI has demonstrated promising potential in differentiating between complex pathological entities, including pancreatic adenocarcinoma, autoimmune pancreatitis, cystic pancreatic lesions, and biliary strictures [9,10].

AI-powered systems—most notably computer-assisted detection (CADe) and computer-assisted diagnosis (CADx)—are emerging as transformative tools. These technologies facilitate real-time lesion detection, classification, and decision support, enhancing both diagnostic accuracy and procedural efficiency. By highlighting suspicious areas for targeted biopsy or further evaluation, AI offers a means to reduce diagnostic oversight and support more timely and precise clinical interventions [11].

Beyond diagnostic enhancements, AI applications in pancreaticobiliary endoscopy are also expanding into procedural guidance and predictive analytics, offering the potential to optimize clinical workflows and improve patient outcomes [1]. As the field continues to evolve, AI-driven solutions are poised to redefine the landscape of pancreaticobiliary diagnostics and therapeutics, advancing toward a more standardized, efficient, and accurate approach to care.

## 2. AI Applications in EUS

EUS is a core modality in pancreatobiliary endoscopy, providing high-resolution imaging of the pancreas, bile ducts, and surrounding structures while also enabling tissue acquisition through fine-needle aspiration (FNA) or biopsy (FNB) [12,13]. It is valuable for detecting a wide spectrum of disorders, including inflammatory conditions such as acute and chronic pancreatitis, cystic lesions such as intraductal papillary mucinous neoplasms (IPMNs) and mucinous cystic neoplasms, and solid pancreatic tumors such as pancreatic ductal adenocarcinoma (PDAC) and neuroendocrine tumors [14,15]. Similarly, EUS facilitates the evaluation of biliary diseases, ranging from benign polyps to malignant lesions such as cholangiocarcinoma [16,17].

Despite its diagnostic utility, EUS faces several important limitations. A large retrospective database evaluation with a real patient cohort reported that the overall diagnostic accuracy ranges from 80% to 95% [18]. For pancreatic cystic lesions (PCLs), accurate risk stratification is critical, but even with adjunctive FNA, EUS achieves only 65–75% accuracy in identifying mucinous lesions. The data is based on a literature review of available case studies, retrospective reviews, and active patient encounters [19]. These diagnostic uncertainties directly influence patient management, particularly given the malignant potential of certain PCLs. While newer modalities such as contrast-enhanced EUS (CE-EUS) and EUS elastography have shown improved specificity—up to 80% for pancreatic cancer [20]—misclassification remains common.

A key contributor to these limitations is operator dependency. EUS is a technically demanding and highly subjective procedure, with diagnostic accuracy varying widely (65–95%) based on experience and expertise [21]. Even in expert hands, interobserver variability persists, with misdiagnosis of subtle lesions still occurring [22]. This challenge is amplified in resource-limited settings, where access to highly skilled operators and advanced equipment is limited [23]. Such variability risks inconsistent diagnoses and missed opportunities for early intervention, particularly for small pancreatic tumors or early biliary obstruction.

**Figure 1:**
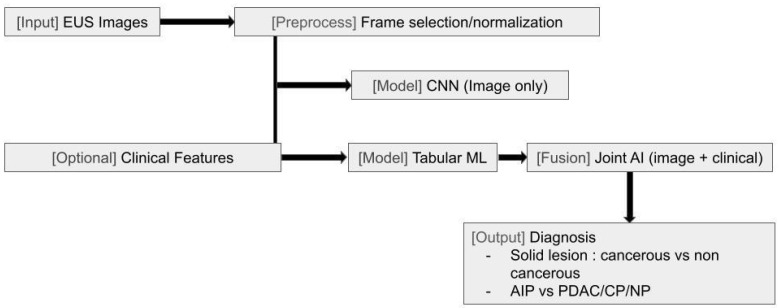
Endoscopic ultrasound (EUS) artificial intelligence diagnostic pathways. Schematic of inputs (EUS images/frames with optional clinical features), preprocessing (frame selection/normalization), model options (image-only convolutional neural network vs. joint image-plus-clinical fusion), and diagnostic outputs (solid lesion classification; autoimmune pancreatitis vs. pancreatic ductal adenocarcinoma/chronic pancreatitis/normal).

Given these challenges, there is a need for strategies to improve consistency and diagnostic accuracy in EUS. Recent advances in machine learning (ML) and deep learning (DL) have introduced new possibilities for EUS image analysis, with early studies demonstrating the potential of AI to aid in differentiating benign from malignant pancreatic lesions and reducing operator dependency [3] (Figure 1 and Figure 4).

One of the major advancements of AI in EUS is its ability to differentiate pancreatic masses that often appear similar on imaging but require vastly different management, such as PDAC, chronic pancreatitis, autoimmune pancreatitis, and pancreatic neuroendocrine tumor (PNET) [24]. This distinction is clinically critical, as PDAC carries a poor prognosis with a 5-year survival rate of only 11% [25]. Several studies have demonstrated AI’s potential to enhance diagnostic accuracy in this setting. A randomized crossover trial in China showed that a multimodal AI model integrating clinical data with EUS imaging significantly improved lesion classification, raising novice endoscopists’ accuracy from 69% to 90% (*p* < 0.001) [26] (Table 1). Similarly, Ozkan et al. developed a CAD system using artificial neural networks that achieved sensitivity, specificity, and accuracy of 83.3%, 93.3%, and 87.5%, respectively [27]. Zhang et al. further demonstrated the value of digital image processing combined with support vector machines, achieving nearly 98% diagnostic accuracy with excellent sensitivity and specificity [28]. Several trials amongst this group are multicenter with successful results in AI-assisted diagnosis. This is attributable to the in silico nature of these works, which removes the variability of real-time endoscopy and relies upon more optimal-quality imaging. However, they serve as a promising step in establishing the validity of AI in the field of EUS.

Beyond PDAC, AI has shown promise in differentiating PNET, which can mimic PDAC, autoimmune pancreatitis (AIP), pancreatic cystic neoplasm (PCN), and even normal pancreas due to overlapping imaging features [29,30]. Ni et al. (2024) [30] developed a CNN model (iEUS) for classifying pancreatic neuroendocrine neoplasm (pNEN) against other pancreatic conditions, achieving accuracies of 84.2% and 88.2% across two classification approaches (binary vs. four-category). Importantly, iEUS outperformed novices and significantly improved sensitivity across all experience levels [30]. Similar to prior studies, a retrospective training arm and a validation cohort were present. Notably, a large sample size and more comprehensive tools would need to be developed to continue to build on this data.

AI has also been applied to distinguish PDAC from chronic and autoimmune pancreatitis, both of which share overlapping imaging characteristics on EUS. Chronic pancreatitis, in particular, poses challenges due to scarring and calcifications that mimic malignancy [31,32]. Early multicenter studies showed that applying artificial neural networks (ANN) to contrast-enhanced harmonic EUS (CEH-EUS) and elastography improved diagnostic performance, with sensitivities and specificities exceeding 85–94%—surpassing even expert endoscopists [32,33,34]. More recently, deep learning approaches have further advanced accuracy. For instance, Kuwahara et al. (2023) used EfficientNetV2-L to classify EUS images of PDAC, autoimmune pancreatitis, and chronic pancreatitis with 91% accuracy, significantly outperforming preoperative diagnosis [18]. Similarly, Marya et al. (2020) applied a CNN (ResNet50v2) that differentiated autoimmune pancreatitis from PDAC, chronic pancreatitis, and normal pancreas with a sensitivity of 90% and a specificity of 85% [31] (Table 1). The data in this study had a longitudinal approach of over two decades, though it was at a single center and retrospective in nature. However, the incorporation of heat block analysis within CNN adds an additional element of future development when AI can be integrated in, giving real-time feedback.

AI has also demonstrated potential in overcoming the diagnostic challenges posed by pancreatic cystic lesions (PCLs). These lesions encompass a broad spectrum of disorders with differing malignant potential. Mucinous cysts, such as intraductal papillary mucinous neoplasms (IPMNs) and mucinous cystic neoplasms (MCNs), are associated with a higher risk of malignancy, whereas non-mucinous lesions, including serous cystic neoplasms (SCNs) and pseudocysts, are typically benign [35,36]. Accurate differentiation between these subtypes is crucial for guiding appropriate management; however, conventional modalities such as EUS and CT show variable sensitivity and specificity and often fail to reliably distinguish mucinous from non-mucinous lesions [37,38].

Kurita et al. (2019) integrated cyst fluid markers and clinical features into a deep learning model that achieved a diagnostic accuracy of 92.9%, with a sensitivity and specificity of 95.7% and 91.9%, respectively—outperforming CEA (60.9%) and cytology (47.8%) [39] (Table 1). Similarly, Boas et al. (2022) developed a CNN trained on 5505 EUS images, reporting an accuracy of 98.5% with an AUC of 1.0 [40] (Table 1). These findings highlight AI’s potential to significantly enhance cystic lesion classification.

Among cystic neoplasms, IPMN is particularly challenging due to its broad spectrum of malignant potential [41,42] (Table 1). Current imaging techniques have limited accuracy in detecting high-risk or malignant lesions [43]. Recent AI-driven approaches have shown encouraging results. Kuwahara et al. (2019) applied ResNet50 to EUS images from 50 IPMN cases, achieving an AUC of 0.98, a sensitivity of 95.7%, a specificity of 92.6%, and an overall accuracy of 94.0%—markedly superior to human diagnosis (56%) [41]. This retrospective study over two decades of data utilized a CNN to evaluate nearly 4000 still images. Complementing this, Machicado et al. (2021) [44] developed CNN-based CAD models for EUS-nCLE, which automated interpretation of features predictive of high-grade dysplasia or carcinoma. In a prospective single-center trial, both segmentation-based and holistic models demonstrated higher sensitivity (83.3%) and accuracy (82.9–85.7%) than guideline-based approaches [44].

Collectively, the advancement of AI in EUS has been remarkable and will only continue to grow. In its current stages, the training and validation across studies is primarily of a retrospective nature and more common in single centers. The available data is largely consistent in showing the benefit of neural networks in providing equivalent if not better accuracy compared to human perception. As AI continues to improve and more real-time data can be collected, further seamless integration will occur.

**Table 1:** Tabular depiction comparing studies.

Modality	Clinical Task	Study (First Author, Year)	Methodology/Model	Design and Setting	Population/*n*	Comparator	Primary Outcome(s)	Key Findings	External Validation
EUS	Carcinoma vs. noncancerous solid pancreatic lesions	Cui, 2024 [26]	Multimodal AI: CNN on EUS + clinical features; multilayer perceptron fusion	Randomized crossover; multicenter (4 centers, China); reader study (12 endoscopists from 9 centers)	Retrospective 628 pts → train/validate 351 (6181 images), internal test 88 (1545); external 189 (1205); prospective crossover 130	Endoscopists with vs. without AI assistance	AUROC: internal 0.996; external 0.955/0.924/0.976 (3 hospitals). External accuracy (Model 3): image 0.84–0.89; patient 0.84–0.91. Reader (novices): accuracy 0.90 with AI vs. 0.69 without (*p* < 0.001); sensitivity 0.91 (95% CI 0.83–0.95).	Multimodal fusion improved generalizability vs. image-only and substantially boosted novice accuracy; minimal benefit for experts; user feedback favored joint-AI	Yes (3 external sets; prospective crossover)
EUS	Autoimmune pancreatitis (AIP) vs. PDAC/CP/normal pancreas	Marya, 2021 [31]	CNN (ResNet-50V2); per-frame and per-video scoring; occlusion heatmaps	Single center (Mayo Clinic, USA); retrospective; internal split by patient	583 pts; 1,174,461 images; test: 974 stills, 376 videos	583 pts; 1,174,461 images; test: 974 stills, 376 videos	Video-only (pairwise): AIP vs. NP Sens 99%/Spec 98%/AUROC 0.992; AIP vs. CP 94%/71%/0.892; AIP vs. PDAC 90%/93%/0.963; AIP vs. all 90%/85%/0.946. Four-class accuracy: CNN 75.6% vs. humans 61.6% (*p* = 0.026).	CNN exceeded experts on four-class diagnosis; interpretable saliency aligned with known EUS features	No (internal only)
EUS	IPMN malignancy (high-grade dysplasia/invasive carcinoma)	Kuwahara, 2019 [41]	CNN (ResNet-50) on pre-operative EUS images	Retrospective; single center; surgical pathology reference	50 patients (benign 27, malignant 23); 3970 EUS still images	Human pre-op diagnosis; mural nodule ≥5 mm; logistic regression	Per-patient AI probability: AUROC 0.98 (vs. mural nodule 0.74, LR 0.73, *p* < 0.001); Acc 94.0%/Sens 95.7%/Spec 92.6%/PPV 91.7%/NPV 96.2% (cutoff 0.41). Per-image AI value: AUROC 0.91; Acc 86.2% (cutoff 0.49).	Deep learning on pre-op EUS stills outperformed human and conventional predictors; AI probability was the only independent predictor of malignancy	No (internal cross-validation only)
EUS	Mucinous vs. non-mucinous pancreatic cystic lesions (PCLs)	Vilas Boas, 2022 [40]	CNN (Xception backbone); internal train/validation	Retrospective; single center; frames from recorded EUS videos	28 pts; 5505 images (train 4404; validation 1101)	Final diagnosis by pathology/cytology/biomarkers; no reader comparator	AUC 1.00; Acc 98.5%/Sens 98.3%/Spec 98.9%/PPV 99.5%/NPV 96.4% (validation).	Single-center pilot CNN accurately differentiated mucinous vs. non-mucinous PCLs; no external or reader comparison	No
EUS	Malignant vs. benign pancreatic cystic lesions	Kurita, 2019 [39]	Deep neural network (2 hidden layers) using cyst-fluid analytes + clinical variables; fivefold cross-validation	Retrospective; single center (Aichi Cancer Center Hospital, Japan)	85 pts (malignant 23, benign 62); sampling: 59 surgery, 26 EUS-FNA	CEA; cytology; AI using CEA only	AI (full inputs): AUC 0.966/Acc 92.9%/Sens 95.7%/Spec 91.9%/PPV 81.5%/NPV 98.3%. CEA: AUC 0.719; Sens 60.9%/Spec 75.8%/Acc 71.8%. Cytology: AUC 0.739; Sens 47.8%/Spec 100%/Acc 85.9%.	AI combining cyst-fluid + clinical features outperformed CEA and cytology, yielding high sensitivity and overall accuracy	No (fivefold cross-validation only)
DSOC	Malignant biliary stricture (MBS) vs. benign	Zhang, 2023 [45]	Two-stage AI: quality-control gating + Vision Transformer (DeiT) classifier	Multicenter; internal/external validation; prospective testing (image and video	Prospective set: image-level; 29 videos; additional internal/external cohorts	Novice/expert endoscopists	Prospective image: Acc 0.923/AUC 0.976. Prospective video: Sens 92.3%/Spec 87.5%.	High performance across internal/external/prospective; video-level accurate; quality-control module essential; outperformed novices/experts; robust across subgroups.	Yes (external + prospective)
DSOC	Neoplasia detection (biliary)	Robles Medranda, 2023 [46]	CNN2; development + multicenter clinical validation	Multicenter clinical validation; prerecorded and real-time DSOC	Frame- and patient-level cohorts; 170 new patients (CNN2 validation)	Expert and non-expert endoscopists	Frame level: Sens 98.6%/Spec 98.0%/PPV 89.2%/NPV 99.2%. Patient level: Sens ~90.5%/Spec ~68.2%/PPV ~74.0%/NPV ~87.8%.	Multicenter DSOC CNN distinguished neoplastic lesions and outperformed non-experts and an expert; applicable to prerecorded and real-time use	Yes (multicenter clinical validation)
DSOC	MBS vs. benign (image level)	Saraiva, 2022 [47]	CNN (image level classifier)	Retrospective, single center (pilot)	85 patients; 11,855 images	None (algorithmic)	5-fold cross-validation: Acc 94.9%/Sens 94.7%/Spec 92.1%/AUC 0.988.	DSOC deep learning accurately discriminated malignant vs. benign; incorporating AI may increase diagnostic yield	No (cross validation only)

## 3. AI Applications in Cholangioscopy and ERCP

### 3.1. (a) AI in Cholangioscopy

**Figure 2:**
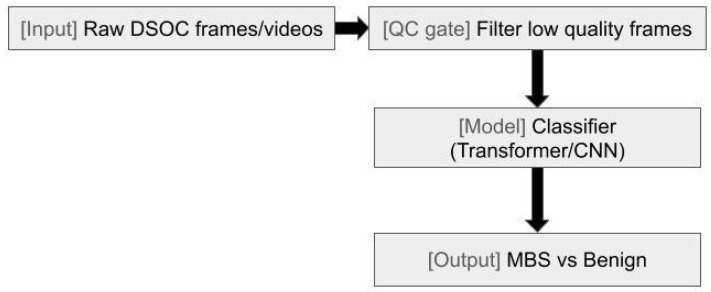
Digital single-operator cholangioscopy (DSOC) diagnostic workflow with quality control. Schematic of raw DSOC frames entering a quality-control gate (low-quality filtering) followed by a classifier (transformer/CNN) to distinguish malignant biliary stricture from benign findings.

Malignant biliary strictures (MBSs) represent a difficult challenge for endoscopists, requiring multiple attempts and often returning with an inconclusive diagnosis. With the introduction of AI into the field, more tools are becoming available for endoscopists to increase yield from cholangioscopy with biopsy and other advanced techniques. Digital single-operator cholangioscopy (DSOC) was first implemented in 2015 to allow high-resolution direct visualization and targeted access to the bile and pancreatic ducts for diagnosing and treating indeterminate biliary strictures, difficult stones, and biliopancreatic neoplasia [48]. While prospective trials have shown increased sensitivity of visualization and overall accuracy utilizing DSOC, room for improvement exists due to interobserver variability and lack of benefit currently in specificity of impression [49,50]. AI tools have been increasingly incorporated to identify macroscopic morphologic characteristics alongside direct visualization to improve lesion identification, stratification, and prognostic prediction (Figure 2 and Figure 4). Currently, three human prospective multicenter trials exist evaluating the efficacy of AI-assisted DSOC with variable results. Two of the studies by Marya et al. and Robles-Medranda et al. utilize CNNs [46,51,52]. CNNs are multilayered neural networks built specifically for imagery analysis via filters whose primary function is to extract necessary features for efficient image processing. Having established precedence from identification of diabetic retinopathy to even polyp detection on endoscopy, CNNs have been proven as effective assistive tools and have been shown to outperform human observers in certain scenarios [53,54].

Looking towards the first study by Marya et al. [52], they performed a two-phase study comparing CNNs in malignant biliary stricture (MBS) identification in comparison to brush cytology and forceps biopsy sampling. The study first retrospectively trained the CNN model on expert analysis from 122 patients with over 14,000 images used for training. This model was then prospectively applied to a cohort of 32 patients on over 5000 images to determine the diagnostic accuracy through occlusion heat block mapping and still-based video analysis [52]. This group was able to demonstrate a higher accuracy on video analysis, above 90%, to that of brush cytology at 63% or even forceps biopsy sampling at 61%. Additionally, the AI determined various features of MBS on heat block mapping. While AI alone would not be sufficient to make the diagnosis, this demonstrates the utility AI could play in real-time analysis or aiding scenarios where sampling returns a non-diagnostic yield. A similar retrospective review by Mascarenhas et al. in Portugal identified a similar diagnostic accuracy of greater than 95% when using a CNN model to when differentiating benign vs. malignant strictures in DSOC [47] (Table 1).

In comparison, Robles-Medranda et al. [46] (Table 1) established a similar two-stage study training the CNN model on pre-recorded videos and real-time DSOC and then trained the same model on additional images based on neoplastic lesion criteria. This model was then compared to a group of experts and non-experts based on randomized and blinded images. The CNN was able to outperform non-experts and one expert [46]. Although variable results compared to endoscopists existed, further growth of the algorithm could lead to successful incorporation of the CNN to real-time procedures.

Zhang et al. differs in that it used data-efficient image transformer (DeiT) in a multicenter study in China [45] (Table 1). DeiT provides advantages utilizing deeper, complex CNNs to transfer knowledge into a simpler model, increasing efficiency while maintaining performance. They applied this two-step version of this model to the identification of MBS, first training this model against thousands of images from multiple centers across China. MBSDeiT would first determine if the image was high-quality and then would perform analysis on characteristics suspicious for neoplasms. They prospectively applied this trained model for real-time performance in 29 patients undergoing DSOC. This group demonstrated an accuracy of 92% on the prospective cohort, establishing itself for use in real-time scenarios [45].

When compared to DSOC without assistance, AI-assisted DSOC enhances the capabilities of the user to better identify lesions, increases diagnostic capability, and can help reduce inter-observer variability. In cholangioscopy, there has been more incorporation of AI models into real-time patient care and more evidence of translation of these validation cohorts. Adding image quality analysis into the network to help further stratify and improve accuracy in real-time scenarios provides another mechanism of support for endoscopists. As the databases continue to expand, larger information pools will be available to train the neural networks to continue to improve their diagnostic accuracy.

### 3.2. (b) AI in ERCP

**Figure 3:**
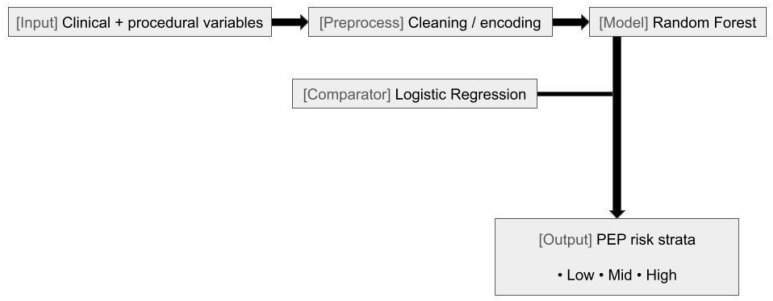
Endoscopic retrograde cholangiopancreatography (ERCP) post-ERCP pancreatitis (PEP) risk prediction workflow. Schematic of inputs (clinical and procedural variables), preprocessing (cleaning/encoding), primary model (random forest) with al logistic-regression comparator, and risk-stratified outputs (low, middle, high).

**Figure 4:**
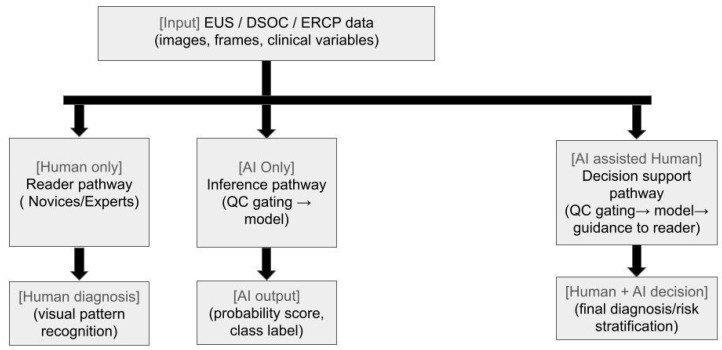
Human versus artificial intelligence (AI) and AI-assisted pathways. Schematic comparison of three decision pathways using endoscopic ultrasound (EUS), digital single-operator cholangioscopy (DSOC), or endoscopic retrograde cholangiopancreatography (ERCP) data: human-only reading (novices/experts), AI-only inference (with quality-control gating), and AI-assisted human decision making.

The choledocholithiasis introduced the need for an improved group of clinical features to identify the presence of choledocholithiasis (CDL) [55]. With the introduction of EUS and MRCP, ERCP has become more limited as a diagnostic option. However, the search remains for a diagnostic tool that is more cost-effective and non-invasive in approach. The rise in AI technology can play an important role in aiding clinicians in making these distinctions (Figure 3 and Figure 4). To assist in the determination of when therapeutic ERCP is needed, Jovanovic et al. relied upon an artificial neural network (ANN) to determine if the combination of non-diagnostic laboratory and imaging tests could accurately predict the need for ERCP [56]. ANNs implement a multilayered approach processing initial data through multiple connected steps to reach an output. A total of 291 patients were evaluated in the study comparing an ANN model to a multivariate logistic regression model, finding the ANN model to have a better discriminant ability and a better accuracy. While more than 80% of patients had positive findings of stones on ERCP and patients were preselected, the study serves as a great proof of concept of the benefits of incorporating AI into clinician-assisting tools [56]. A retrospective review by Huang et al. [57] sought to compare a CNN model against non-expert and expert endoscopists for assessing the difficulty of stone extraction in ERCP. Trained on over 1000 images based on criteria of various stone and anatomical characteristics, the AI was able to outperform non-expert endoscopists and was comparable to endoscopists in rating difficulty of stone extraction. These more challenging stone extractions were often associated with a need for endoscopic papillary large-balloon dilation or lithotripsy [57].

Another area AI has been integrated into ERCP includes reducing radiation exposure during procedural fluoroscopy. Bang et al. [58] performed a prospective study of 100 patients who underwent conventional versus AI-assisted fluoroscopy during ERCP. The AI system decreased exposure by reducing the radiation only towards areas of interest for endoscopists with real-time adjustment [58]. The model relies on a CNN as described above to limit shutter exposure, trained on over 10,000 images. In demographic matched cohorts of 50 patients, the AI-assisted fluoroscopy led to a significant reduction in radiation exposure and scatter compared to that of conventional fluoroscopy, with exposure of 2178 mGy m2 (IQR 1308.6–4317.7) vs. 5708 mGy m2 (IQR 1965.7–7953.3), *p* = 0.001 [58]. Not only was the AI able to lead to improved occupational safety, but the system also correctly identifies the region of interest within the field, allowing for easier maneuvering for endoscopists.

Machine learning (ML) has even been applied to evaluating predictors for post-ERCP pancreatitis (PEP), one of the most common complications advanced endoscopists face. A retrospective trial in Japan sought to identify predictors and risk-stratify patients undergoing ERCP to reduce PEP. A total of 40 patient characteristics were identified on patients undergoing ERCP, comparing a machine learning algorithm to a linear regression model to determine factors associated with PEP. The data established the ML model as superior to logistic regression in predicting PEP as well as regarding risk stratification [59]. It represents a powerful tool that when developed further could be able to enhance patient care preventing adverse events.

The current literature surrounding ERCP and AI has not had as much growth compared to EUS and cholangioscopy. Available data largely centers around predictive risk factors for procedural complications in retrospective neural network assessment. Continued expansion is needed in terms of datasets, training cohorts, and progression to prospective evaluations.

## 4. Benefits of AI

As shown above, AI can transform current care models to become even more efficient and reduce adverse events.

One of the hardest challenges faced when standardizing endoscopy is the inter-observer variability, especially when looking at less objective findings. Whether this represents tasks such as measuring the lengths of Barrett’s epithelium or classifications of inflammatory bowel disease, variability amongst trainees and experts is always present [60,61]. Even when experts have fewer differences in interpretation, room for further standardization always exists. Introduction of AI models in real-time endoscopy can further reduce deviations in diagnosis and therapy. Looking towards implementation of CNNs to DSOC, one can see the increase in diagnostic accuracy in identifying MBS as well as identifying characteristics concerning malignancy. Comparing this type of model to endoscopists was shown to be equivalent or occasionally superior to expert endoscopists. Workflow efficiency is further enhanced, given MBS biopsies and brush cytology often yield non-diagnostic results. Real-time analysis will help to reduce the need for repeat interventions and would allow for quicker access to treatment. Even when looking at ANNs in EUS identifying pancreatic cancer or CNNs for differentiating AIP from PDAC, diagnostic accuracy increases, and time to identification decreases. Having an expert-equivalent model available in real time will reduce variability amongst trainees and experts alike while enhancing workflow.

Not only does AI enhance outcomes, but it can be used to help decrease adverse events. ML algorithms were able to better identify characteristics associated with PEP compared to current models. Furthermore, AI-assisted fluoroscopy was able to decrease radiation exposure during ERCP. Both outcomes demonstrate the ability of AI to reduce harm in acute settings as well as longitudinally.

Integration into endoscopic platforms may help standardize image interpretation, reduce operator variability, and shorten procedure times [16,21,23]. As mentioned above, AI assistance improved accuracy in identifying MBS, differentiating pancreatic lesions, and reducing radiation exposure during ERCP [26,31,45,52,58] can decrease the need for repeat procedures and enable earlier, more accurate diagnosis, thereby improving patient outcomes. From a health economic perspective, enhanced diagnostic efficiency may reduce downstream costs associated with delayed diagnosis, redundant testing, and unnecessary interventions [1,55,56,58].

Even in novel fields such as EUS, ERCP, and DSOC, AI has started to show the potential for significant improvements. These models can continually grow as neural networks build upon their training. Progressive use of DeiTs as seen on pre-existing datasets could create perpetual enhancement of efficiency and performance as more data is processed. With continued advancement, new areas of integration will develop, and AI will play a more integral role in endoscopy. The next steps involve directly integrating many of these models into video processors or endoscopies, reducing the cost of computer-aided interventions, and increasing the use of current datasets. Real-time interpretations and automated landmark identification would accelerate workflows and reduce cognitive load. As AI expands into other facets, domains such as pathology, genetics, and future AI genomics, future systems are expected to integrate imaging, clinical, and molecular data to advance personalized diagnostics and care. As these tools become embedded within endoscopic hardware and reporting platforms, their costs are likely to decline, enabling scalable, cost-effective implementation even in resource-limited settings [1,16,23].

## 5. Limitations and Challenges

AI for pancreatobiliary endoscopy is in its early stages [1,16]. Unlike polyp detection in colonoscopy, where several systems are already commercially available, AI software for EUS, ERCP, and cholangioscopy is still in the form of research prototypes [1,16]. To our knowledge, there are no FDA cleared or authorized AI systems available for these indications [62]. Most reported models are retrospective, single center, with scant external, multicenter validation, such that routine clinical use is premature [1,16,63].

The core issue is data. EUS/ERCP volumes are significantly lower than routine luminal endoscopy, leading to small, typically single-center training datasets [1,16,63]. Datasets may be enriched for surgical cases or lack lookalike benign disease (e.g., chronic pancreatitis), thus narrowing what the model learns and generalizability. For example, a tumor vs. normal classifier trained primarily on surgical cancers may mislabel chronic pancreatitis nodules on real world EUS since it never “saw” those lookalikes in training [63].

Acquisition and hardware heterogeneity affect performance. Differences in scope vendors, ultrasound probe frequencies, image processors, and capture settings modify image texture and noise. Without standardized acquisition and labeling, models overfit one environment and do not generalize well elsewhere [1,63]. For instance, a model that is fine-tuned on a 12 MHz radial EUS dataset may fail on a 7.5 MHz linear probe; lesion borders are different even when the pathology is the same. National and international archives, with harmonized metadata, capture protocols, and labels, are needed to train on diverse, interoperable videos [1,16].

External, multicenter validation remains limited. Many systems report good internal metrics but drop when validated across devices and sites. As a concrete example, a single-center area under curve (AUC) of nearly 0.90 may drop to ~0.75 when evaluated across hospitals with different processors and workflows—good enough to publish, but not good enough to deploy without guardrails [1,16].

Governance and privacy are also limitations on progress. Endoscopy generates large video streams, and pooling data between institutions raises consent, privacy, and cross border transfer questions. Federated learning and auditable, secure data exchange systems can but are yet to be widely adopted solutions [1,64,65]. Model interpretability remains a barrier to trust. Deep learning systems tend to be “black boxes.” Clinicians need meaningful explanations and calibrated confidence/uncertainty in order to know when to trust a prediction and when to be wary of it [1,63].

Finally, implementation is everything. Even useful models break down if they do not fit in the workday. Systems must interface with recording/reporting workflows and Picture Archiving and Communication System/Electronic Health Record (PACS/EHR), and teams need practical training on how to operate, interrogate, and override AI. Current curricula are still catching up [1].

To summarize, science is promising, but large, standardized data and prospective, multicenter validation, as well as privacy preserving collaboration and clinician-friendly interfaces, are the bridge from prototype to practice [1,16].

## 6. Future Applications 

### 6.1. (a) Future Directions

Delivering on AI’s promise in pancreatobiliary endoscopy will require better data, stronger methods, and careful implementation. The first priority is scale and diversity. Recent reviews emphasize the need for national or international repositories of annotated EUS/ERCP images and video, so models learn across institutions, devices, and patient populations, not just one center’s experience [1,16]—for example, a pan-national EUS/ERCP video repository containing deidentified clips and harmonized metadata (probe type, frequency, processor, and diagnosis) with tiered access for research and benchmarking. These repositories must conform to FAIR principles and assume harmonized capture and labeling protocols in order to improve portability [1]. Where end-to-end pooling is not feasible, federated learning and auditable sharing schemes may allow cooperation without raw data transfer; GI endoscopy reports expressly name federated learning as privacy preserving training [64]. For example, each hospital locally trains and sends only batches of model updates; a central server pools them and proposes a new model to all sites. Issues of privacy and governance with massive video stores also support secure, auditable exchange mechanisms [65].

Second, we must increase the evidentiary bar. Future, multicenter trials with open reporting should be standard. CONSORT-AI and SPIRIT-AI provide reporting and protocol standards required to prespecify the outcome and assess performance systematically [66,67]. As endoscopy units run on varied hardware, cross-platform testing, both vendor to vendor and processor to processor, is essential to ensure that a model trained in one setting will work in another [1]. For example, a classifier trained by Vendor A is prospectively tested on Vendors B and C before clinical release. In the case of devices that will alter, the FDA now provides a path via Predetermined Change Control Plans (PCCPs) such that AI-based devices can update safely after clearance [68]—for example, a PCCP enabling periodic retraining on new cases. Combining endoscopic images with clinical, laboratory, and molecular data should improve risk prediction and individualize therapy [1]. Multicenter early experience with digital single-operator cholangioscopy shows the manner in which size and heterogeneity improve diagnosis of malignant stricture and serves as a model for larger datasets [69].

Big language models and multimodal networks in the future will be capable of making high-level decisions and automating reporting using text, pictures, and audio/video [16]. These models should show the reason behind a prediction, e.g., saliency/attention maps and calibrated confidence, to build trust and enable safety checks (CONSORT-AI) [66]. For example, the user interface refers to a suspicious rim, calls to mind parallel past cases, and possesses an 0.86 confidence, thus recommending biopsy now or short interval follow-up. Ultimately, workflow and workforce create world impact. Tools must work seamlessly with endoscopy platforms and the EHR, so advice is at the point of care without extra clicks, and teams need coordinated training to use, query, and, as needed, override AI [1,16]. With common datasets, prospective multicenter evaluation, interpretable design, and transparent regulatory channels, AI can move from promising prototype to trusted day-to-day aid in EUS and ERCP.

### 6.2. (b) Future Clinical Scenarios

AI has the potential to transform clinical decision-making in pancreatobiliary endoscopy by seamlessly integrating multimodal data to guide individualized care. In EUS, the ability of DL algorithms to differentiate pancreatic adenocarcinoma from inflammatory or neuroendocrine lesions in real time would optimize decisions on biopsy necessity or surgical referral [18,23]. For pancreatic cystic lesions, AI models that combine imaging, molecular markers and cytology may accurately predict malignancy risk, enabling personalized surveillance or timely resection [19,39,41,70]. During ERCP, AI-assisted digital cholangioscopy may detect subtle MBS, reducing diagnostic uncertainty [46,51,69]. Additionally, AI-driven fluoroscopy analysis could dynamically guide cannulation strategies while minimizing radiation exposure [58]. As these systems evolve, AI may not only improve diagnostic accuracy but also predict therapeutic outcomes, ultimately supporting a fully customized, data-driven approach to pancreatobiliary care.

## 7. Costs of AI Adoption in Endoscopy

The costs associated with AI adoption in endoscopy departments, including hardware, software, training, and maintenance, are increasingly relevant as AI applications expand to modalities such as endoscopic ultrasound (EUS), ERCP, and cholangioscopy.

Hardware costs involve upgrading endoscopy processors and imaging platforms to support AI integration, which may require high-performance computing units and compatible video capture devices. For pancreatobiliary endoscopy, this often means additional investment in digital cholangioscopy systems and EUS processors capable of real-time AI analysis [24,70,71].

Software expenses are typically incurred through licensing or subscription fees for AI algorithms tailored to imaging, such as lesion detection, stricture characterization, and risk prediction. These costs can vary depending on the vendor and complexity of the AI, with some platforms charging per procedure or per device [24,70,72].

Training costs are significant, as endoscopists and staff must be educated on the use of AI tools, workflow integration, and interpretation of AI-generated outputs. This includes initial onboarding and ongoing education as software updates, and new functionalities are released [32,72].

Maintenance costs encompass regular software updates, hardware servicing, and technical support to ensure compliance, data security, and optimal performance. These are recurring expenses and may be bundled with software licensing or charged separately [70,72].

While initial investment is significant, AI adoption may improve diagnostic accuracy, workflow efficiency, and long-term cost-effectiveness through earlier detection and intervention [24,70]. However, the real-world cost data for AI adoption remains limited, and further evaluation is needed to guide scalable implementation.

## 8. Ethical Considerations for AI Use in Endoscopy

Key ethical considerations for AI in pancreatobiliary endoscopy include algorithmic bias, data privacy, transparency, liability, and effects on the clinician–patient relationship. Bias may arise from non-representative datasets, necessitating diverse, high-quality data and ongoing validation to ensure equitable care [72,73,74]. Protecting patient privacy and ensuring informed consent are critical, particularly for secondary data use, requiring robust governance [73,74,75]. Transparency and explainability are essential for clinician trust and informed decision-making, with professional societies emphasizing interpretable algorithms [72,74,75]. Clear liability frameworks are needed for diagnostic errors or adverse events, while careful integration of AI should enhance, not undermine, patient autonomy and trust [73,74,75]. Finally, the impact on the clinician–patient relationship must be considered. Over-reliance on AI may risk undermining patient autonomy and trust, while appropriate integration can enhance shared decision-making and care quality [74,75].

## 9. Conclusions

AI is shifting from promise to practice in pancreatobiliary endoscopy. Some of the most potent research already shows what is possible: an early ANN model helped triage patients for therapeutic ERCP; an AI assisted fluoroscopy system reduced radiation in ERCP; and a multicenter digital single-operator cholangioscopy study separated malignant biliary strictures with strong performance. They tell us that, with excellent data and design, AI can spot easily missed patterns and automate difficult steps in care [56,58,69].

In the meantime, the evidence base is still evolving. Most pancreatobiliary models are based on small, single-center, low external validation retrospective cohorts. Systematic reviews in 2025 make the same case for pancreatic cysts. As of July 2025, there are no FDA-approved AI systems for EUS, ERCP, or cholangioscopy (in contrast to colonoscopy CADe). Regulation and science agree here: we need broader, real-world evidence before routine use [1,24,62,76].

GastroNet-5M is a cloud-based endoscopy image library containing ~5 million anonymized images from 500,000 procedures across multiple centers, supporting AI model development for general and pancreatobiliary endoscopy [77]. Other public datasets, such as HyperKvasir and EPIC, offer large, multi-class image collections, though coverage of pancreatobiliary procedures is more limited [78,79]. Reviews indicate over 30 publicly accessible endoscopic image libraries, most focusing on luminal GI pathology rather than specialized pancreatobiliary content [80]. ASGE consensus statements emphasize that multimodal, high-quality datasets and cloud-based tools are essential for advancing AI in gastroenterology. However, large, annotated datasets for specialized pancreatobiliary applications, including EUS, ERCP, and cholangioscopy, remain scarce, highlighting the need for further resource expansion [32,70,71,72]. Moreover, large, publicly accessible image repositories are commonly available for training AI models in radiology but not in gastrointestinal endoscopy because radiology imaging is standardized and routinely archived in digital formats (DICOM), whereas endoscopic imaging is highly heterogeneous, operator-dependent, and lacks uniform labeling and annotation standards, making large-scale, high-quality datasets much more difficult to collect and share [78,80,81,82].

The path ahead is obvious and practical. Create shared, well-annotated repositories for EUS/ERCP; normalize capture and labels, and use federated learning when data cannot move. Perform prospective, multicenter open reporting and cross-platform tests. Interpret models and multimodal models and integrate them into the endoscopy platform and EHR so advice is at point of care. Train staff to apply and overrule AI. And while AI will not replace the endoscopist, it will be a valuable partner that identifies disease earlier, guides safer intervention, and individualizes treatment [1,16,24,69].
